# Perturbed maintenance of transcriptional repression on the inactive X-chromosome in the mouse brain after Xist deletion

**DOI:** 10.1186/s13072-018-0219-8

**Published:** 2018-08-31

**Authors:** Robin L. Adrianse, Kaleb Smith, Tonibelle Gatbonton-Schwager, Smitha P. Sripathy, Uyen Lao, Eric J. Foss, Ruben G. Boers, Joachim B. Boers, Joost Gribnau, Antonio Bedalov

**Affiliations:** 1 Clinical Research Division, Fred Hutchinson Cancer Research Center, 1100 Fairview Avenue N., Seattle, WA 98109 USA; 2 Department of Biochemistry, University of Washington, Seattle, WA 98195 USA; 3 Department of Medicine, University of Washington, Seattle, WA 98195 USA; 4 Department of Developmental Biology, Erasmus MC, 3015 CN Rotterdam, The Netherlands; 5 Department of Obstetrics and Gynaecology, Erasmus MC, 3015 CN Rotterdam, The Netherlands; 6 Delft Diagnostic Laboratories, 2288 ER Rijswijk, The Netherlands

**Keywords:** X-chromosome inactivation, Xist, Noncoding RNA, MeCP2, Rett syndrome

## Abstract

**Background:**

The long noncoding RNA Xist is critical for initiation and establishment of X-chromosome inactivation during embryogenesis in mammals, but it is unclear whether its continued expression is required for maintaining X-inactivation in vivo.

**Results:**

By using an inactive X-chromosome-linked MeCP2-GFP reporter, which allowed us to enumerate reactivation events in the mouse brain even when they occur in very few cells, we found that deletion of Xist in the brain after establishment of X-chromosome inactivation leads to reactivation in 2–5% of neurons and in a smaller fraction of astrocytes. In contrast to global loss of both H3 lysine 27 trimethylation (H3K27m3) and histone H2A lysine 119 monoubiquitylation (H2AK119ub1) we observed upon Xist deletion, alterations in CpG methylation were subtle, and this was mirrored by only minor alterations in X-chromosome-wide gene expression levels, with highly expressed genes more prone to both derepression and demethylation compared to genes with low expression level.

**Conclusion:**

Our results demonstrate that Xist plays a role in the maintenance of histone repressive marks, DNA methylation and transcriptional repression on the inactive X-chromosome, but that partial loss of X-dosage compensation in the absence of Xist in the brain is well tolerated.

**Electronic supplementary material:**

The online version of this article (10.1186/s13072-018-0219-8) contains supplementary material, which is available to authorized users.

## Background

X-chromosome inactivation in female mammals equalizes the autosome to X-chromosome gene dosage in males (XY) and females (XX) [1]. Two waves of X-inactivation are observed during early mouse embryogenesis, an extensively studied model for X-inactivation in mammals (reviewed in [2, 3]). An initial wave of inactivation occurs at the 2–4-cell stage in the embryo, specifically on the paternal X-chromosome (Xp) [4, 5]. This imprinted X-inactivation pattern remains throughout development in the extraembryonic tissues, which give rise to the trophectoderm and primitive endoderm, but it is reversed at a late blastocyst stage in the inner cell mass (ICM) [6], which forms the embryo proper. Following reactivation of the Xp, a second wave of inactivation in the ICM after implantation (E 5.5–6.5) randomly inactivates either the maternal X (Xm) or Xp [5, 6], generating a mosaic inactivation pattern that is clonally propagated and maintained through the lifetime of the animal.

The process of X-inactivation can be divided into four stages: initiation, spreading, establishment and maintenance [2]. X-inactivation initiates with the expression of the long noncoding RNA, Xist (X-inactive-specific transcript), from the chromosome to be inactivated [7, 8], which spreads in *cis* along the entire chromosome and recruits additional silencing factors, which together establish stable repression of that chromosome for the lifetime of the cell and its progeny (reviewed in [9]). Among repressive factors recruited to the Xi by Xist are the polycomb repressive complexes 1 and 2 (PRC1 and PRC2), which catalyze monoubiquitylation of lysine 119 on histone H2A (H2AK119ub1) and trimethylation of lysine 27 on histone H3 (H3K27me3) ([10], reviewed in [11]), respectively, and many others identified through different oligonucleotide pull-down screens [12–14]. DNA methylation is thought to be a later event in the process of X-inactivation [15]. The expression of Xist continues during the maintenance phase, generating a cloud of Xist that surrounds the Xi.

The essential role of Xist and dosage compensation in mouse embryonic development has been established by genetic analysis of Xist mutations. Xist-loss-of-function mutations inherited from the father lead to early, female-specific, embryonic lethality due to loss of imprinted X-inactivation and failure to establish XCI in the extra embryonic tissues [16]. A poorly developed trophectoderm, which fails to supply the conceptus with the necessary nutrients, is thought to be the primary reason for the embryonic lethality in females lacking paternal Xist, given that the X-chromosome inactivation in the inner cell mass is preserved due to inactivation of the Xm with intact Xist [5, 6]. In contrast to the inheritance of paternal Xist mutation, which is lethal, maternal Xist mutation in female embryos is tolerated because imprinted inactivation in the extraembryonic tissues is preserved and because XCI in the embryo proper, while infeasible on the Xm, can be accomplished by skewed inactivation of the Xp [5, 6, 16]. Yang et al. [17] recently deleted Xist specifically in the inner cell mass, circumventing the essential role of Xist in the extraembryonic tissues and early embryonic lethality, to further examine the role of Xist in establishment of random X-inactivation and the role of dosage compensation during development. Even though these animals fail to establish a properly inactivated Xi, displaying partial loss of dosage compensation, organogenesis was grossly normal with many pups surviving to term. However, all the animals succumb by 3 weeks of age, consistent with decreased overall fitness due to X to autosomal imbalance. These results together revealed a critical role for Xist during initiation and establishment of both imprinted and random X-inactivation.

While the critical role of Xist in initiation and establishment of X-chromosome inactivation is well supported by mouse genetic studies, the role of Xist in the maintenance of X-chromosome inactivation is not as clearly defined. Deletion of Xist in the mouse hematopoietic compartment during the maintenance phase leads to highly penetrant leukemia, consistent with an important role for Xist in suppressing hematologic malignancies [18]. Furthermore, two unbiased genetic screens using X-linked reporter genes on the Xi identified Xist and Xist regulators as factors required for maintaining the silenced state on the Xi [19, 20]. However, other studies examining X-reactivation in human or mouse differentiated cells found no requirement for Xist in maintaining repression on the Xi. Even though expression of Xist as well as Xist cloud formation is normally present during the maintenance phase, genetic ablation of Xist in mouse fibroblasts, without concomitant DNA demethylation, did not result in appreciable Xi reactivation [21]. Similar conclusion that both Xist deletion and DNA demethylation are needed for Xi reactivation was drawn in a recent study that employed global transcriptional profiling across the X-chromosome in the mouse brain [22]. Different outcomes of these studies, which might reflect the use of different model system or methods for measuring X-reactivation, make it difficult to draw a conclusion about the role of Xist in maintenance of Xi.

To clarify the role of Xist in the maintenance of random X-inactivation, we examined the effect of deletion of Xist on the Xi in the developing mouse brain using a Nestin-Cre driver [23], expressed during the time window after random Xi has been well established. Critically important for this study, because we suspected that reactivation might occur in only small fraction of cells, we employed a MeCP2-EGFP reporter gene on the Xi, which is driven by the endogenous MeCP2 promoter known to be highly active in neurons when on the Xa [24]. We found that deletion of Xist leads to nearly complete loss of H3K27m3 and H2AK119ub1, consistent with the proposed role of Xist in the recruitment of PRC1 and PRC2 to the Xi [11]. However, despite loss of both Xist and these repressive histone modifications, the MeCP2 reporter gene on the Xi was reactivated in only 2–5% neurons. Animals tolerated this partial loss of dosage compensation in the brain surprisingly well, without increased mortality, fertility or obvious neurologic defects. Our results unequivocally demonstrate that continued expression of Xist plays a role in maintaining faithful transcriptional repression of the Xi in vivo but that partial loss of dosage compensation in the absence of Xist in the brain is well tolerated.

## Results

**Fig. 1:**
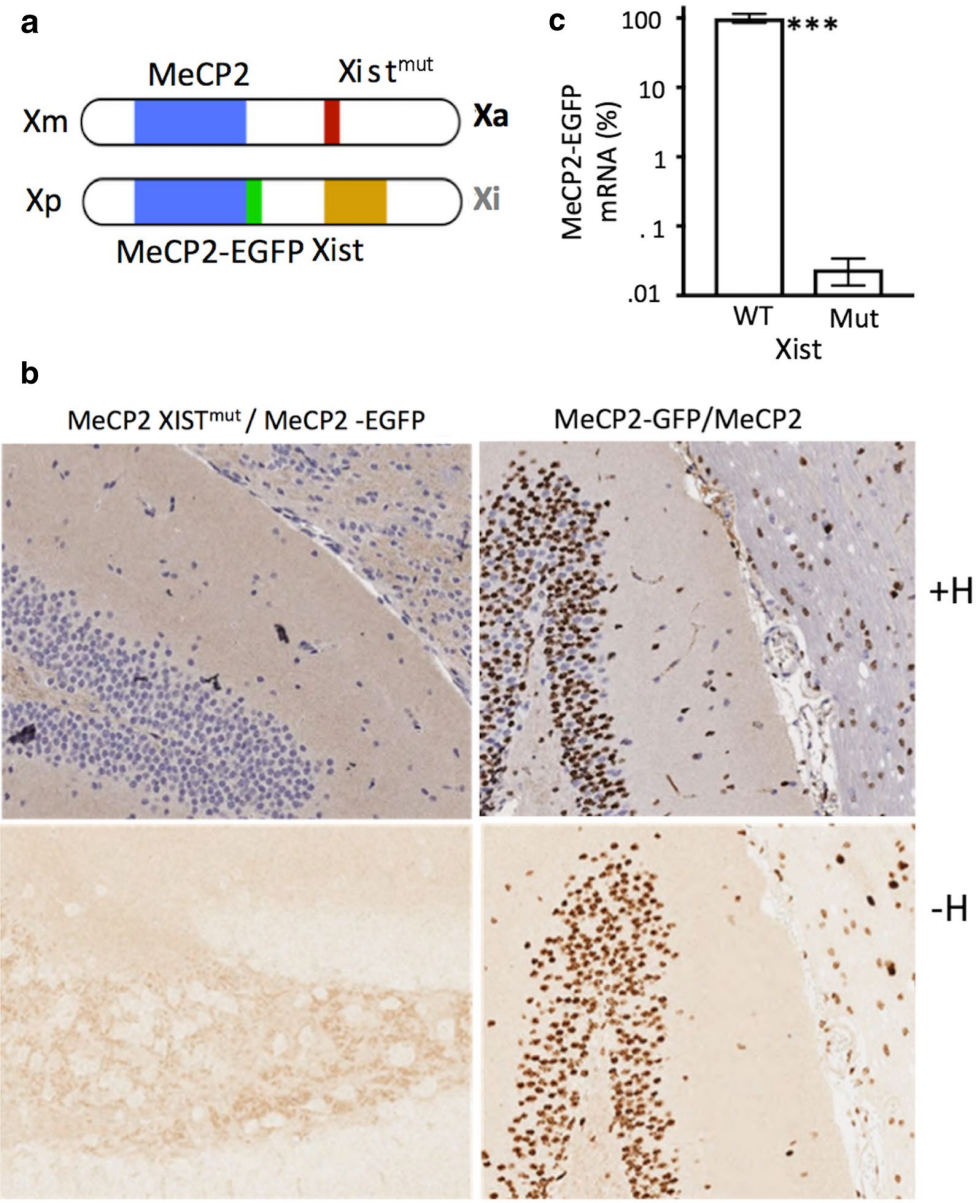
**a** Mutation of Xist on the maternal X-chromosome leads to skewed inactivation of the MeCP2-GFP reporter gene on the paternal X-chromosome. **b** GFP immunohistochemistry with and without hematoxylin (+H and −H) in the brain of the MeCP2 Xist^mut^/MeCP2-GFP female demonstrates no GFP staining. The brain of the MeCP2/MeCP2-EGFP animal exhibits a bimodal nuclear staining for GFP reflective of random MeCP2-EGFP inactivation. **c** MeCP2-EGFP mRNA level in the brain from MeCP2 Xist^mut^/MeCP2-EGFP relative to MeCP2-EGFP/MeCP2 analyzed by RT-qPCR (*n* = 3 for each group, error bars indicate SD, ***p < 0.001, Student’s *t* test)

As discussed previously, female mice that inherit maternal Xist-loss-of-function mutations exhibit exclusive inactivation of the Xp. We exploited this X-inactivation pattern to generate a model in which an X-linked MeCP2-EGFP reporter gene is uniformly silenced in all cells. We employed two Xist-loss-of-function mutations: Xist^fl^, containing a deletion of the promoter and the first three exons of Xist [25], and Xist^tm5Sado^, containing a deletion of the proximal A repeat of Xist, which causes loss of Xist expression [26]. As both of these alleles were previously shown to confer inability to inactivate X-chromosome and yielded the same results in our studies, we refer to them collectively as Xist^mut^. We crossed females heterozygous for Xist^mut^ with males carrying EGFP reporter fused at the C terminus to MeCP2 to obtain (Xist^mut^ MeCP2) maternal (m)/(Xist MeCP2-GFP) paternal (p) females (Fig. 1a). While MeCP2-EGFP is highly expressed throughout brain of MeCP2-GFP/MeCP2 females, we found no expression in (Xist^mut^ MeCP2)m/(Xist MeCP2-GFP)p females (Fig. 1b), consistent with uniform silencing of the MeCP2-GFP transgene on the Xp. RT-qPCR using primers that amplify the EGFP transcript confirmed these histology results (Fig. 1c). Given complete skewing of X-chromosome inactivation and thus absence of MeCP2-GFP expression, we felt that this model would be sufficiently sensitive to detect low levels of the reporter gene reactivation that would be obscured in a MeCP2/MeCP2-GFP animal, in which large fraction of cells expresses MeCP2-GFP as baseline.

We used this experimental system to determine whether Xist is required in vivo for maintaining the silenced state of MeCP2-GFP on Xi in brain after silencing has already been established. After introducing the floxed Xist allele (Xist^2lox^) [25] to the X-chromosome carrying MeCP2-EGFP reporter gene [24], we crossed the hemizygous males carrying this X-chromosome MeCP2-GFP-Xist^2lox^/y) with heterozygous Xist/Xist^mut^ females that also carry the Nestin-Cre transgene to obtain Xist^mut^/MeCP2-GFP-Xist^2lox^ females with and without the Nestin-Cre transgene [23]. Cre-mediated recombination of the Xist^2lox^ transgene deletes the promoter and first three exons of the Xist gene. Random X-inactivation occurs between embryonic days (E) 5.5 and 6.5, whereas the Nestin-Cre is expressed in neuronal lineages peaks at E 12.5 with little to no detectable expression before E 9.5 [23, 27]. Therefore, the female progeny carrying Nestin-Cre would excise the paternal Xist2lox allele after random X-inactivation has been established, and the progeny without Nestin-Cre transgene would serve as littermate controls.

**Fig. 2:**
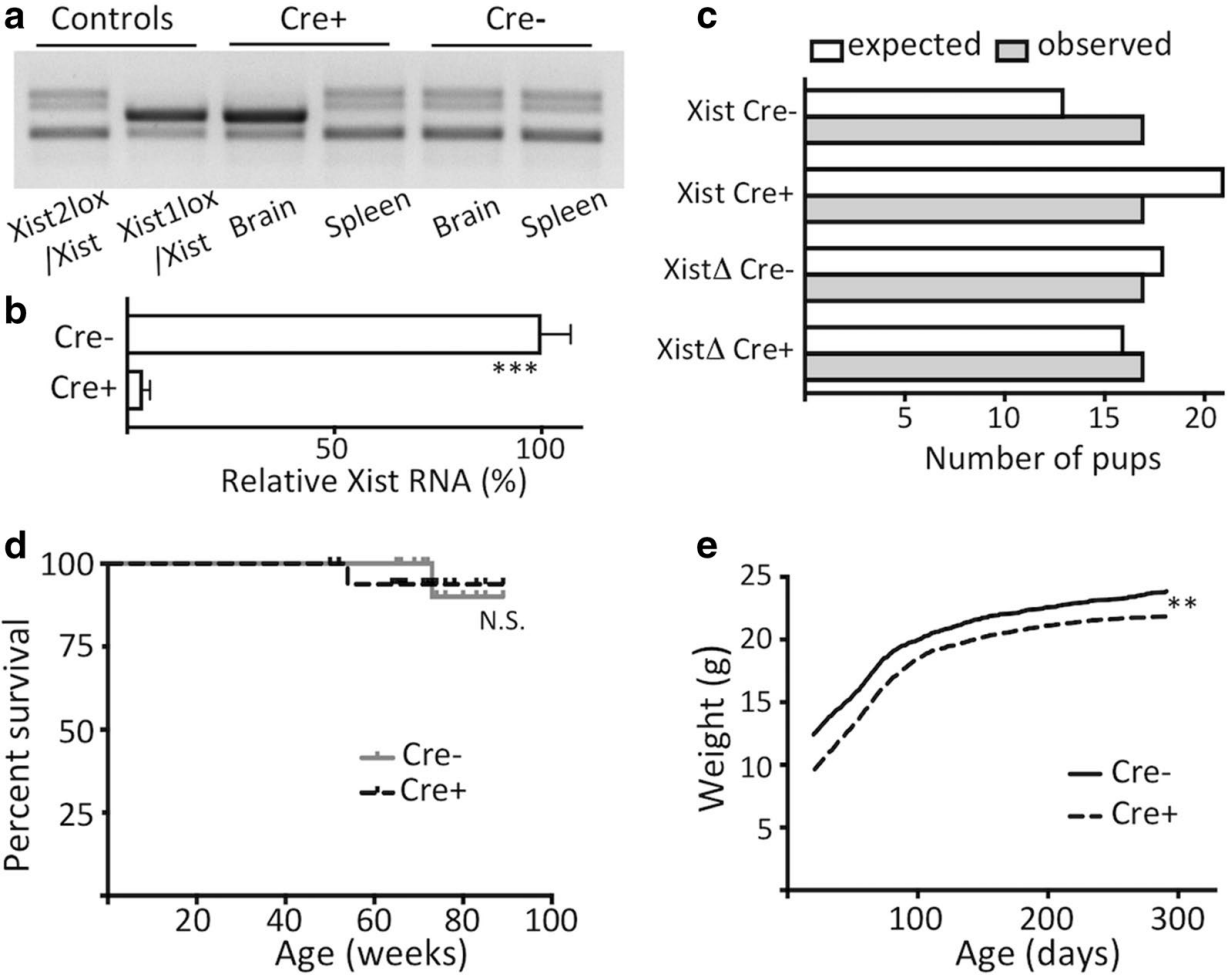
**a** Nestin-Cre deletes Xist^2lox^ allele in the brain but not in the spleen of Xist^mut^/MeCP2-GFP-Xist^2lox^ female animals. Ethidium bromide-stained gel of PCR products for Xist in the DNA extracted from the indicated tissues in animals with (Cre+) and without (Cre−) Nestin-Cre transgene. **b** Xist RNA level measured by RNA-seq in the RNA extracted from the Xist^mut^/MeCP2-GFP-Xist^2lox^ females with (Cre+) and without (Cre−) Nestin-Cre transgene (*n* = 3). **c** Xist^mut^/ Xist^2lox^ Nestin-Cre females that lack Xist in the brain were born at the expected Mendelian frequency (*p* = 0.36, Chi-square test). **d** Survival curves of females with Nestin-Cre transgene (nes +) and their littermates without (Cre−) the transgene (Cre+ *n* = 18, Cre− *n* = 17, N.S. *p* = 0.58, log-rank test). **e** Two-week sliding averages of weights in Xist^mut^/MeCP2-GFP Xist^2lox^ females with (Cre+) and without (Cre−) Nestin-Cre transgene (Cre+ *n* = 14, Cre− *n* = 15, (**p < 0.01, Student’s *t* test, for all successive 2-week periods for the duration of the follow-up)

We examined DNA extracted from brain and spleen from Nestin-Cre mice and found complete excision in the brain and no excision in the spleen (Fig. 2a), consistent with the known pattern of Nestin-Cre expression [23]. In Nestin-Cre negative control littermates, we observed no excision in either of these tissues. RNA-Seq data for Xist transcript levels revealed > 20-fold decrease in Xist abundance in the RNA extracted from the brains of Nestin-Cre animals compared with their littermate controls without Nestin-Cre transgene (Fig. 2b). Xist^mut^/ Xist^2lox^ Nestin-Cre females that lack Xist in the brain were born at the expected Mendelian frequencies (Fig. 2c) and did not exhibit any anatomical defects, and their survival was indistinguishable from negative control littermates during the follow-up of up to 20 months (Fig. 2d). Furthermore, they successfully bred and gave birth to litters of the usual sizes.

The only phenotype we observed in animals carrying Nestin-Cre transgene was reduced weight and body size evident throughout their lifespan (Fig. 2e). To determine whether the weight deficit in Nestin-Cre animals is dependent on the excision of the Xist transgene in Xist^mut^ /Xist^2lox^ females, we crossed Xist^mut^/Xist females, with males heterozygous for Nestin-Cre and compared weights of Xist^mut^/Xist females with and without Nestin-Cre. The weight deficit of the Nestin-Cre animals compared with their littermate controls in the cohort of animals without the floxed Xist allele (XIST^mut^/XIST, Additional file 1 lower panel) was similar to the deficit in the cohort of floxed Xist animals (XIST^mut^/XIST^2lox^, Additional file 1 upper panel), 2.2 g versus 1.75 g, respectively, at weaning, and 1.2 g versus 1.8 g at 3 months. Animals with Nestin-Cre transgene had comparable weights whether they also have or do not have the XIST^2lox^ transgene, 7.8 ± 0.4 g versus 8.3 ± 0.3 g (*p* = 0.23, Student’s *t* test), respectively, at weaning and 18.4 ± 0.3 g versus 18.6 ± 0.2 g (*p* = 0.48 by Student’s *t* test), respectively, at 3 months. Similar reduction in weight and body size, attributed to transgene-induced hypopituitarism and dwarfism, has been previously reported for Nestin-Cre transgenic animals [28]. We conclude that the reduced body weight in the Xist^mut^ /Xist^2lox^ Nestin-Cre animals is caused by the Nestin-Cre transgene and not by the deletion of Xist.

**Fig. 3:**
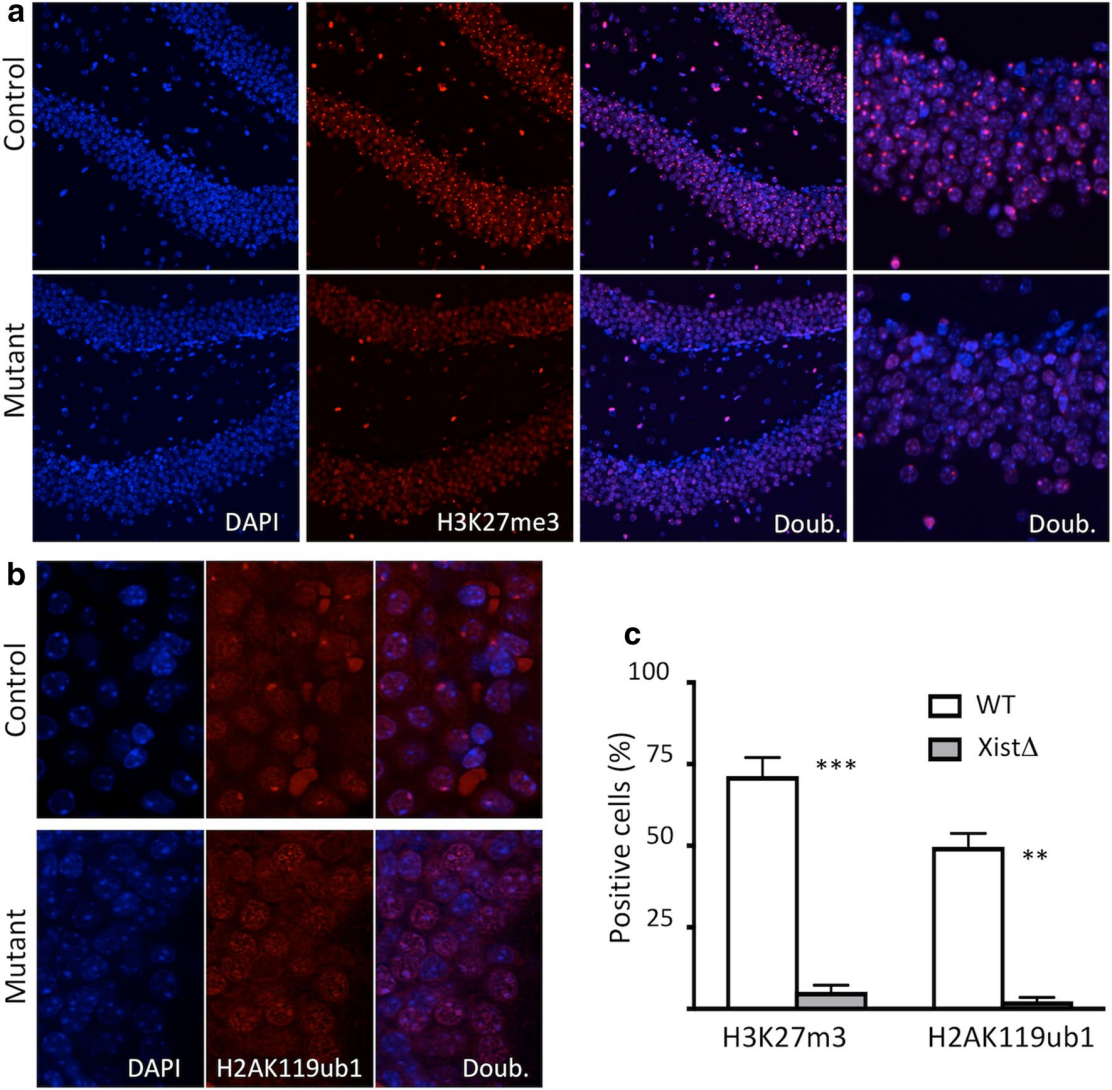
IF for **a** H3K27me3 and **b** H2AK119ub1 in the brain of animals without (control) and with Xist deletion (mutant). Punctate nuclear staining for both H3K27me3 and H2AK119ub1 is abolished by Xist deletion. **c** Percentage of cells in the brain with punctate staining for the indicated histone modification is severely reduced in animals lacking Xist (*n* = 3 for each group, error bar indicates SD, **p < 0.01, ***p < 0.001, Student’s *t* test)

Xist is required for the recruitment of PRC1 and PRC2, responsible for monoubiquitylation of lysine 119 on the histone H2A (H2AK119ub1) and trimethylation of lysine 27 on histone H3 (H3K27me3) on the Xi, respectively, which are critical for the establishment of XCI [11]. To examine whether the maintenance of these repressive histone marks depends on Xist expression, we compared immunofluorescence (IF) signal for both H2AK119ub1 and H3K27me3 in brain tissues from Cre+ and Cre− animals. As expected, the control Cre− littermates, with intact Xist, exhibited punctate staining at the nuclear periphery for both H3K27me3 and H2AK119ub1 and (Fig. 3a, b), marking the inactive X-chromosome in large fraction of cells, 72 ± 6% for H3K27me3 and 49 ± 4% for H2AK119ub1, along with diffuse staining throughout the nucleus. In Nestin-Cre animals, punctate staining pattern for both histone marks was lost in the vast majority of cells while the diffuse staining was maintained (Fig. 3a–c). We conclude that Xist is required for the maintenance of both H2AK119ub1 and H3K27me3 repressive marks on the Xi.

**Fig. 4:**
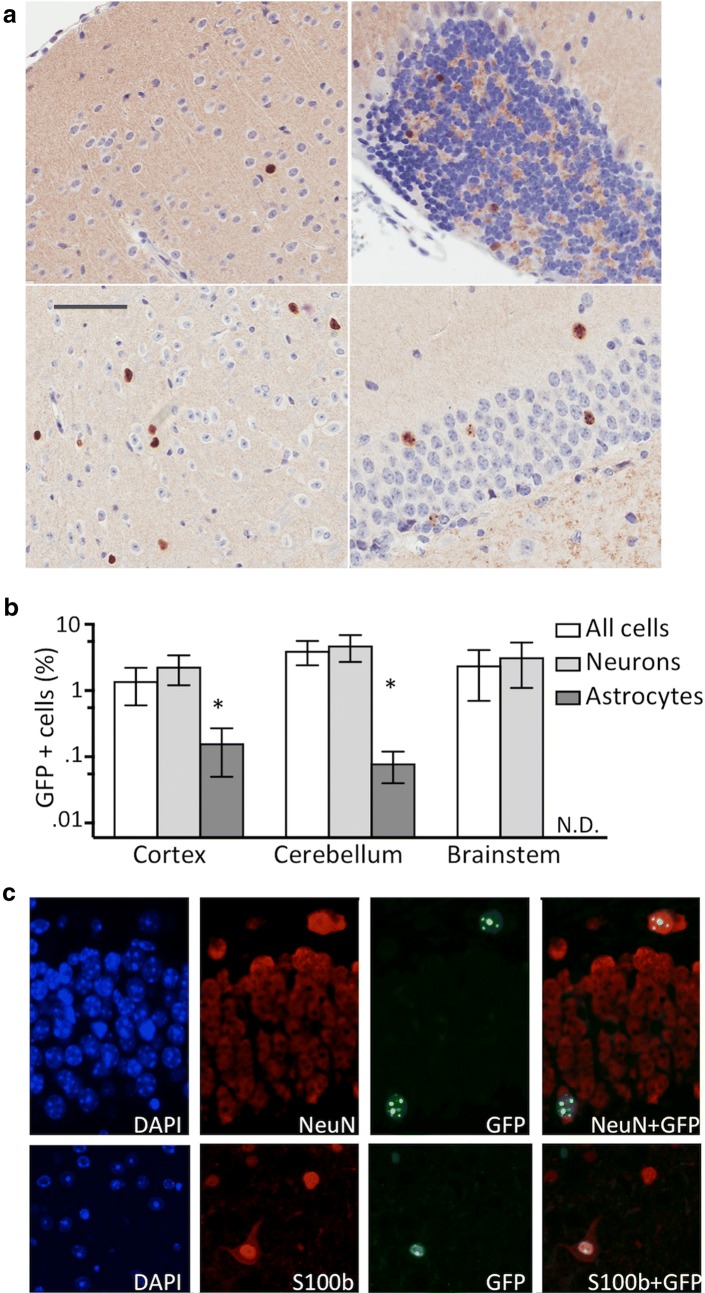
**a** GFP IHC in different brain regions including cortex, hypocampus, brainstem and cerebellum (clockwise starting from left upper panel) of Nestin-Cre+ Xist^mut^/MeCP2-GFP Xist^2lox^ animals lacking Xist in the brain. Bar indicates 80 µm. **b** Percentage of GFP-positive cells in the indicated brain regions in all cells, NeuN-positive neurons and S100b-positive astrocytes (*n* = 4, error bar indicates SD, neurons vs astrocytes, *p < 0.05, Student’s *t* test). **c** Reactivation of MeCP2-GFP reporter gene in neurons and astrocytes in brains from Nestin-Cre+ Xist^mut^/MeCP2-GFP Xist^2lox^ animals. Double IF for GFP and NeuN (upper panel) and for GFP and S100b (lower panel) demonstrates GFP IF in both NeuN-positive neurons and S100b-positive astrocytes

To determine whether deletion of Xist is required for maintaining MeCP2-GFP silencing, we performed immunohistochemistry (IHC) for GFP on the brain tissue from mice with and without Nestin-driven Cre, alongside positive control females (MeCP2-EGFP/MeCP2-EGFP) and wild-type control females. Whereas no GFP-positive cells were observed in the brains of the Nestin-Cre negative controls, we found that 1.4–4.0% of cells in the brain parenchyma express GFP (Fig. 4a, b). To determine the cell types that express MeCP2-GFP, we performed double IF stains for GFP with anti-NeuN and with anti-S100b antibodies, marking neurons and astrocytes, respectively (Fig. 4c). The vast majority of EGFP-positive cells also expressed NeuN, demonstrating MeCP2-GFP reactivation in neurons. While the reactivation of MeCP2 was not limited to neurons, as we also observed GFP-positive cells among the S-100b expressing astrocytes, the fraction of astrocytes that express GFP was lower compared to neurons (0.1–0.2% vs 2.3–4.8%, *p* < 0.05, Student’s *t* test) (Fig. 4b, c). EGFP expression level in MeCP2 Nestin-Cre animals measured by qPCR was 3.2 ± 0.8% of the expression observed animals with the MeCP2-GFP reporter gene on the Xa, which is similar to the results observed by IHC or IF (Additional file 2A).

**Fig. 5:**
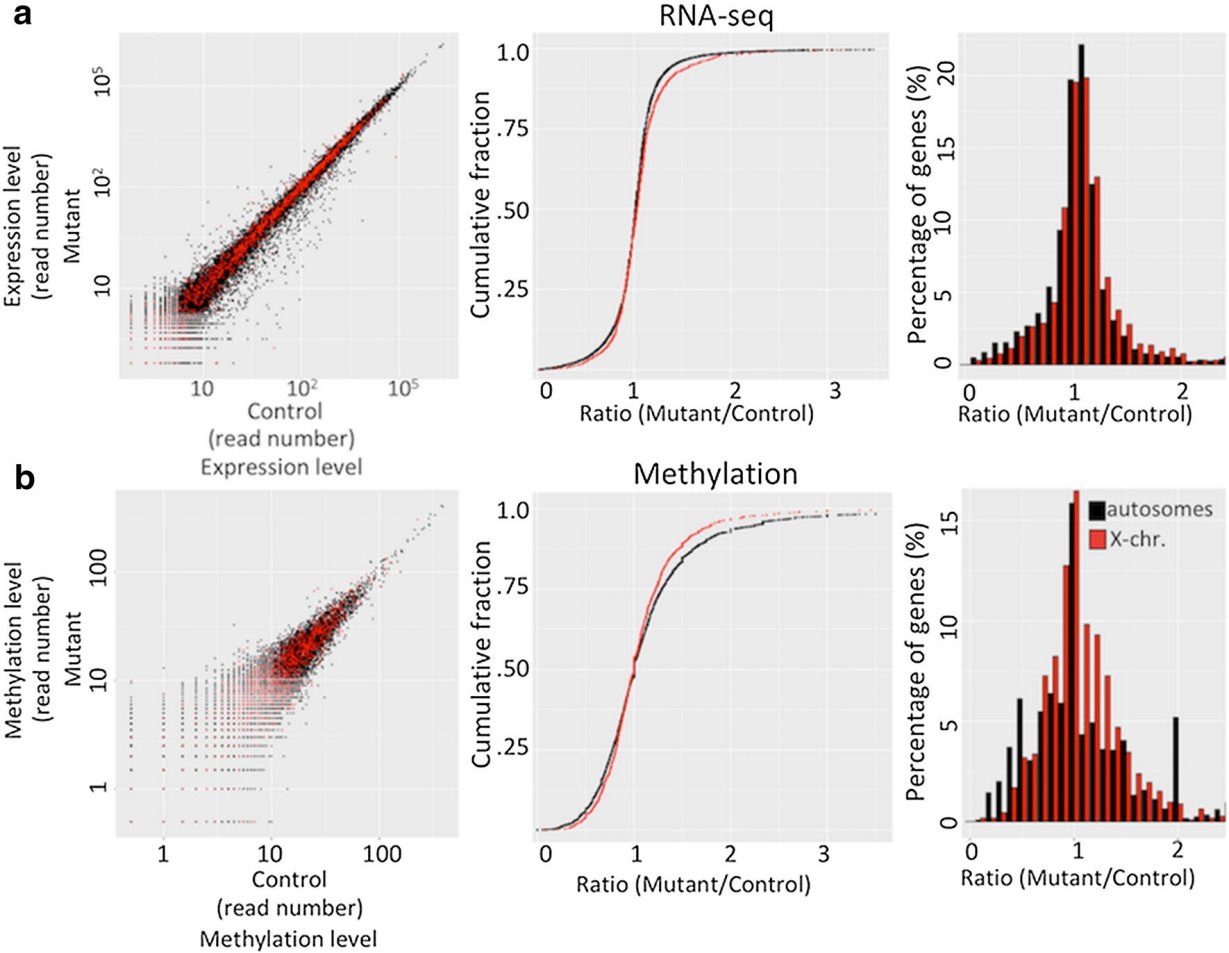
Analysis of **a** global transcriptional and **b** DNA methylation changes on X-chromosome and autosomes in animals lacking Xist. **a** Left: Scatter plot of RNA expression levels for genes autosomes (black) and X-chromosome (red) (*N* = 3 for both mutant and control animals). The genes on X-chromosome exhibit an upward shift relative to autosomes. Middle: Cumulative expression plots of fold expression changes (mutant/control) for genes on autosomes (black) and X-chromosome (red). The genes on X-chromosome exhibit a rightward shift (*p* < 0.01, Wilcoxon rank-sum test). Right: Binned fold changes in expression for genes on autosomes (black) and X-chromosome (red). **b** Left: Scatter plot of DNA methylation abundance at CpG islands on autosomes (black) and X-chromosome (red) (*N* = 2 for both mutant and control animals). The CpG islands on X-chromosome exhibit a downward shift relative to CpG islands on autosomes. Middle: Cumulative plots of fold methylation abundance changes (mutant/control) for CpG islands on autosomes (black) and X-chromosome (red) (*p* < 0.01, Wilcoxon rank-sum test). Right: Binned fold changes in methylation abundance at CpG islands on autosomes (black) and X-chromosome (red)

The pattern of expression of MeCP2-GFP in Nestin-Cre animals is bimodal, as judged by IHC and IF, with cells either fully expressing or not expressing the reporter gene, suggesting that silencing of the MeCP2-GFP remains intact in a large fraction of cells and that dosage compensation is largely preserved for this gene despite the absence of Xist. To examine the consequences of Xist deletion on expression level of other genes on the X-chromosome, we compared the RNA expression level in brains by RNA-seq in Nestin-Cre-positive animals and their Nestin-Cre-negative control littermates. While gene expression scatter plots for genes on the autosomes did not differ between animals lacking Xist and control animals, the scatter plots of the genes on the X-chromosome exhibited an upward shift compared to autosomes (Fig. 5a left panel, Additional file 2B), suggesting that genes on the X-chromosome were overexpressed in the mutants. Cumulative expression plots of the fold expression change in the mutant compared to the controls for genes on the X-chromosome exhibited a subtle but significant shift to the right compared to the autosomes (*p* < 0.01, Wilcoxon rank-sum test) (Fig. 5a middle panel, Additional file 3). Similar comparison of control RNA samples showed no difference between X-linked and autosomal genes (Additional file 2C). When we analyzed binned fold expression changes (Fig. 5a right panel) of the same data, we observed a subtle shift to the right, averaging 0.04, in the expression level relative to the expression levels on the autosomes upon Xist deletion. We conclude that deletion of Xist leads to an increase in the expression of genes on the X-chromosome but the magnitude of the overexpression was subtle, suggesting that other genes are reactivated in only small fraction of cells, similarly to what we have observed with MeCP2.

**Fig. 6:**
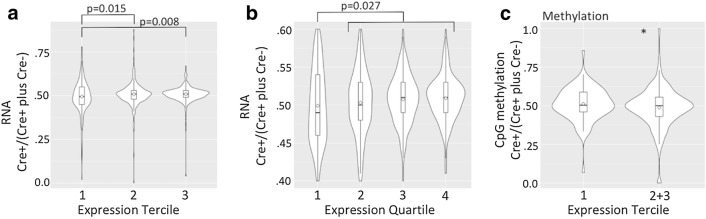
Violin plots of the ratios of the RNA reads in Cre+/Cre+ plus Cre−) animals for X-linked genes that are divided into terciles (**a**) and quartiles (**b**) according to their expression level. Each tercile and quartile has 180 and 135 genes, respectively. All *p* values were calculated using Student’s *t* test. The average ratios for quartiles 1–4 are 0.494, 0.504, 0.508 and 0.510, respectively. The differences among adjacent quartiles are not significant. **c** Changes in CpG island methylation expressed as a ratio of methylation abundance in Cre+/(Cre+ plus Cre−) animals for genes grouped according to their expression level, as in (**a**), with the first tercile compared with an aggregate of the second and the third terciles (*p = 0.045, Student’s *t* test, 0.487 ± 0.133 vs 0.512 ± 0.109, *N* = 180 first tercile, *N* = 360 combined second and third terciles)

As already shown in Fig. 4b, we observed that the level of reactivation of the MeCP2-GFP reporter in neurons is approximately one order of magnitude higher compared to the level of reactivation in astrocytes (2–5% vs 0.1–0.2%). Thinking that this might reflect the higher level of MeCP2 expression in neurons compared to astrocytes, we hypothesized that, after transcriptional silencing is perturbed by deletion of Xist, more potent transactivation associated with highly expressed genes might lead to their more frequent reactivation on the Xi compared to genes with low expression levels. To test this idea, we divided the genes on the X-chromosome into terciles based on their expression level, after excluding the genes that are covered with fewer than 20 sequencing reads whose low expression level precludes accurate measurements, and compared the level of reactivation among the terciles. The median level of expression in the third tercile was similar to the expression level of MeCP2, while the expression level in the second and first terciles was approximately tenfold and 100-fold lower, respectively. The level of gene reactivation was measured as a fraction of reads in Cre+ samples over total reads (Cre+ plus Cre−), which is expected to be > 0.5 if Xist deletion causes loss of repression on the Xi. The fraction of reads in Cre+ samples for genes in the third tercile was higher compared to genes in the first tercile (0.509 ± 0.047 vs 0.495 ± 0.096, *p* = 0.008, Student’s *t* test), which corresponds to an average increase of 3% in the expression level for the genes in the third tercile, similar in magnitude to the level of reactivation we observed for MeCP2. The middle tercile exhibited a reactivation similar to the third tercile and higher than the first tercile (0.508 ± 0.047 vs 0.495 ± 0.096, *p* = 0.015, Student’s *t* test) (Fig. 6a). Similar results were obtained when the genes were divided into quartiles, with the genes from the second to fourth quartiles exhibiting a progressive increase in reactivation level (Fig. 6b). These results demonstrate that genes on the Xi that are normally highly expressed are more prone to reactivation upon deletion of XIST than genes with lower expression levels, with highly expressed genes exhibiting a similar magnitude of reactivation as we observed for MeCP2.

We next examined whether Xist deletion changes DNA methylation on the X-chromosome. We used the methylation-dependent restriction enzyme LpnP1 to interrogate almost 50% of all CpG sites across the genome, as described [29]. CpG island methylation on autosomes, examined by scatter plots, did not differ in brains from mutants and their wild-type littermates, whereas the scatter plots of CpG islands on X-chromosomes exhibited a subtle downward shift (Fig. 5b left panel, Additional file 2D). This finding suggests overall decreased methylation on the X-chromosome upon Xist deletion. Cumulative distribution plots of fold changes in methylation of CpG islands demonstrated that deletion of Xist reduced methylation in a small fraction of CpG islands but also increased methylation levels at even smaller faction of other CpG islands, with the predominant overall shift toward hypomethylation (*p* < 0.01, Wilcoxon rank-sum test) (Fig. 5b middle and right panels, Additional file 4). These results suggest that Xist is required for faithful maintenance of DNA methylation on the Xi and that its loss creates overall decrease in DNA methylation of the X-chromosome.

Having observed (1) that Xi exhibits demethylation and (2) that highly expressed gene is more prone to reactivation upon Xist deletion, we wondered whether the extent of demethylation, likewise, correlates with the gene expression level. To test this idea, we compared changes in methylation level on the CpG islands located within 4 kb of the transcription start site for the genes in the first tercile of the expression level, which as a group did not exhibit derepression, with the composite of the second and third tercile, both of which exhibited derepression. We observed that the genes in the later group had on average 5% reduction in the methylation level compared to the former (0.487 ± 0.133 vs 0.512 ± 0.109, *p* = 0.045, Student’s *t* test) (Fig. 6c). These results demonstrate that the higher level of transactivation associated with highly expressed genes is also reflected in a higher level of demethylation of their CpG islands on the Xi.

## Discussion

In this study, we used Nestin-Cre to delete Xist specifically on the Xi in the developing mouse brain after silencing has been established, and measured reactivation of a MeCP2-EGFP reporter gene on the Xi. The Xm carrying an Xist mutation and a wild-type allele of MeCP2, combined with the Xp, which carried both a floxed Xist allele and the MeCP2-EGFP reporter, ensured that the reporter gene and the floxed Xist allele are uniformly on the Xi. The lack of detectable expression of MeCP2-EGFP reporter in the brain enabled detection of reactivation in even a small fraction of cells upon Nestin-Cre-mediated excision of Xist. Because Nestin-Cre-mediated recombination in neural progenitors occurs well after XCI is established, our study addresses the role of Xist during the maintenance phase. We found that H2AK119ub1 and H3K27me3 modifications are largely abolished upon deletion of Xist. However, despite the loss of both Xist and these histone repressive marks, only 1.4–4.0% of all cells in brain parenchyma, or 2.3–4.8% of neurons, exhibit reactivation of the MeCP2-EGFP reporter gene.

The pattern of MeCP2-EGFP expression upon loss of Xist is bimodal, with most cells exhibiting either full or no detectible expression. This result demonstrates that MeCP2 silencing is maintained in a large fraction of cells either because X-inactivation is intact along the entire chromosome in a majority of cells, and the small fraction of cells that express MeCP2-EGFP have lost silencing along the entire chromosome, or because loss of silencing occurs only in fragments of the Xi in a larger fraction of cells. While we cannot differentiate between these two possibilities given a single reporter in our study, we favor the latter because different loci on the Xi likely differ in their propensity to lose transcriptional repression, with genes that are highly expressed more prone to reactivation compared to genes with lower expression level. We believe that the higher level of MeCP2-EGFP reactivation observed in neurons compared to astrocytes is more likely a reflection of higher promoter activity and expression of MeCP2 in the neurons compared to astrocytes, leading to increased reactivation at the MeCP2-EGFP locus in neurons, than an intrinsic cell-type difference in the ability to keep X-inactivation intact along the entire chromosome. This reasoning is supported by our observation presented in Fig. 6AB that X-linked genes that are highly expressed are more prone to reactivation compared to genes with lower expression level.

Many of the repressive factors are recruited to the Xi via their interaction with Xist. Among these factors are PRC1 and PRC2, which are responsible for deposition of the H2AK119ub1 and H3K27m3 repressive marks on the Xi [10, 11]. Our observation that these two histone modifications are largely abolished upon Xist deletion supports the idea that the continued presence of Xist at the Xi is required for maintaining PRC1 and PRC2 and the associated enzymatic activities at the Xi. However, as silencing of the MeCP2 reporter gene was intact in the vast majority of cells, it is likely that other factors, once recruited to Xi, no longer require Xist to remain on the Xi and enforce transcriptional repression. Dnmt1 has been identified as another factor that is physically tethered to Xi by Xist [14]. Consistently, we found a decrease in DNA methylation on the X-chromosome upon deletion of Xist in the brain. However, these methylation changes were subtle compared to global loss of H2AK119ub1 and H3K27m3 repressive marks. Largely preserved methylation on the Xi makes this modification a likely candidate for the maintenance of Xi in the brain in the absence of Xist. This possibility is also supported by the previously published in vitro study demonstrating that while deletion of Xist alone resulted in no reactivation, deletion of Xist combined with either small molecule inhibition of DNA methyltrasferases or a mutation in Dnmt1 resulted in a robust reactivation of the reporter gene [21], and by the recent mouse study that reported X-chromosome-wide expression changes when combining Xist deletion with pharmacologic inactivation of DNA methyltrasferases [22]. These results suggest that DNA methylation maintains transcriptional repression of the Xi in the absence of Xist and that DNA methylation at the Xi during maintenance phase is largely independent of Xist.

We show that deletion of Xist leads to subtle changes in methylation of CpG islands on the X-chromosome as compared to CpG islands on the autosomes. The magnitude of these changes was small, similar to what we have observed with the transcriptome. Furthermore, the extent of gene demethylation at the Xi, similar to derepression, correlated with the gene expression level, which supports the idea that higher level of transactivation associated with highly expressed genes makes these genes more prone to both derepression and demethylation on the Xi. Similar observation that transcriptional activators can overcome position effect variegation has been reported at telomeric loci in *S. cerevisiae* [30]. Interestingly, while the transcription from the X-chromosome was uniformly increased upon Xist deletion as compared to autosomes, consistent with reduced transcriptional activation on the Xi, CpG island methylation changes were bidirectional, with some islands demonstrating hypomethylation, and a smaller fraction exhibiting hypermethylation in response to Xist deletion. Bidirectional changes in CpG islands methylation during transcriptional activation on the Xi are not surprising because of the known association of transcription with hypomethylation of promoter CpG islands and hypermethylation of intragenic CpG islands [31]. Indeed, X-chromosome inactivation escaper genes are characterized by CpG island hypomethylation of their promoters and hypermethylation in their gene bodies [32].

Despite partial loss of dosage compensation, animals lacking Xist in the brain appeared healthy, had no increased mortality during the follow-up of up to 2 years and were fertile delivering the usual litter size. It is widely believed that preserving dosage balance between autosomes and sex chromosomes is critical for development, organismal viability and fitness across different species regardless of the dosage compensation strategy employed to achieve the balance [33]. In support of this view, genetic perturbation of dosage compensation mechanisms in fruit flies (male sex lethal), nematodes or mice creates sex-specific embryonic lethality. Our result which addresses specifically the maintenance of X-inactivation in the mouse brain, as well as recent studies focusing on the establishment of random X-inactivation during embryogenesis [17], challenges this view, suggesting instead that perturbation of dosage compensation in mice is tolerated better than previously thought.

## Conclusions

The importance of Xist for initiation and establishment of X-chromosome inactivation and X-chromosome dosage compensation during embryogenesis has been well established through mouse genetic studies. Our study demonstrates that Xist also plays a role in the maintenance of DNA methylation and transcriptional repression on the inactive X-chromosome in vivo during phase but that partial loss of X-dosage compensation upon Xist deletion in the brain, with reactivation in 2–5% of neurons, is well tolerated. The tolerance of partial loss of X-chromosome dosage compensation in the brain in our study raises the prospect for therapeutic reactivation of MeCP2 or other genes in Rett syndrome and other X-linked genetic disorders in females.

## Methods

### Mice

Mouse strain carrying Xist^tm5Sado^ allele (B6;129-Xist^tm5Sado^, Riken Bioresource Center), with a deletion in the Xist proximal A repeat [26], was obtained from the Disteche Laboratory at the University of Washington with the permissions from the Riken BRC and Dr Takashi Sado, who has donated the strain to the Riken BRC. The Xist^2lox^ mouse strain (129-Xist^tm2Jae^/Mmnc, stock number 29172-UNC) was obtained from the Mutant Mouse Regional Resource Center (MMRRC), a NIH-funded strain repository, and was donated to the MMRRC by Rudolf Jaenisch, Ph.D., Whitehead Institute. Mice strains carrying Nestin-Cre (B6.Cg-Tg(Nes-Cre)1Kln/j, stock number 003771) and MeCP2-EGFP transgenes (MeCP2^tm3.1Bird^/J, stock number 014610) were obtained from the Jackson Laboratory. All animals were maintained on a C57BL/6 background. Animal work was carried out according to the National Institute of Health and institutional guidelines and was approved by the Fred Hutchinson Cancer Research Center Institutional Animal Care and Use Committee.

### RNA analysis

Total RNA was extracted from the brains of 2–3-month-old animals using Trizol LS Reagent (Thermo) using manufacturer instructions. RNA integrity was checked using an Agilent 2200 TapeStation (Agilent Technologies, Inc., Santa Clara, CA) and quantified using a Trinean DropSense96 spectrophotometer (Caliper Life Sciences, Hopkinton, MA).

RNA-seq libraries were prepared from total RNA using the TruSeq RNA Sample Prep Kit v2 (Illumina, Inc., San Diego, CA, USA). Library size distribution was validated using an Agilent 2200 TapeStation (Agilent Technologies, Santa Clara, CA, USA). Additional library QC, blending of pooled indexed libraries and cluster optimization were performed using Life Technologies’ Invitrogen Qubit^®^ 2.0 Fluorometer (Life Technologies-Invitrogen, Carlsbad, CA, USA). RNA-seq libraries were pooled (8-plex) and clustered onto two flow cell lanes. Sequencing was performed using an Illumina HiSeq 2500 in rapid mode employing a paired-end, 50 base read length (PE50) sequencing strategy. Image analysis and base calling were performed using Illumina’s Real Time Analysis v1.18 software, followed by “demultiplexing” of indexed reads and generation of FASTQ files, using Illumina’s bcl2fastq Conversion Software v1.8.4.

For RT-qPCR, RNA was extracted as above, and cDNA was prepared using iScript™ Reverse Transcription Supermix for RT-qPCR (BioRad) as per the manufacturer’s instructions. Quantitative PCR (qPCR) was performed using Prism 7900HT (Applied Biosystems), with Sso Advanced Universal SYBR Green Supermix (BioRad). PCR conditions were set up as per the manufacturer’s instructions using primers listed in Additional file 5. qPCR values were normalized to GAPDH values using the ΔΔCt method.

### DNA methylation analysis

Methylation analysis was carried out using methylation-dependent restriction enzyme LpnP1 as described [29]. Briefly, DNA, extracted from the brains of 2–3-month-old female mice using phenol–chloroform (Sigma-Aldrich) and digested with LpnP1, was used to generate sequencing libraries. Because Lpn1 cleaves the DNA at a fixed position (N12/N16) downstream from the methylated cytosine, a symmetrically methylated CpG dinucleotide is expected to generate an approximately 32-bp fragment. Pippin Prep (Sage Science, Boston, MA) gel system was used to enrich for the fragments of the desired size, and the size-selected library was sequenced. The pipeline for processing of sequencing data, LpnP1 filter validation and data analysis was carried out as we described previously [29].

### Immunohistochemistry

Brains were removed, fixed in 10% formalin and embedded in paraffin. Four-micron coronal brain sections were stained with “Leica Bond Rx” (Leica Biosystems, Buffalo Grove, IL). Slides undergoing GFP analyses were pretreated with H2 antigen retrieval buffer for 35 min. Endogenous peroxidase was blocked with 3% hydrogen peroxide for 5 min. A TCT protein block was applied for 10 min (0.05 M Tris, 0.15 M NaCl, 0.25% Casein, 0.1% Tween 20, pH 7.6) and incubated with Anti-GFP antibody (1:1000, Invitrogen; A11122) for 60 min. All antibodies were then detected using Leica Power Vision HRP rabbit-specific polymer (Leica Biosystems; DS9800) for 12 min. Nuclear-specific staining was visualized with “Refine DAB” (Leica Biosystems; DS9800). A hematoxylin counterstain was then applied. Isotype negative control antibody-exposed slides were included as controls.

### Immunofluorescence

Brains were removed, fixed in 10% formalin and embedded in paraffin. Four-micron coronal brain sections were stained with “Leica Bond Rx” (Leica Biosystems, Buffalo Grove, IL). For NeuN and GFP dual IF staining, slides were pretreated with H1 antigen retrieval buffer for 20 min. Avidin/biotin block (Biocare; AB972M) was applied for 10 min each followed by 10-min incubation in TCT protein block (0.05 M Tris, 0.15 M NaCl, 0.25% Casein, 0.1% Tween 20, pH 7.6). Anti-NeuN (1:400, Millipore; MAB377) biotinylated with goat anti-mouse Fab (Jackson 115-067-003) was used for 60 min followed by SA-Alexa Fluor 568 (1:200, Invitrogen, catalogue no. 511226) at 1:200 for 30 min. Another sequential immunofluorescence procedure was followed directly after repeat avidin/biotin and TCT protein blocks as described above using anti-GFP (1:25, Invitrogen; A11122) for 60 min and followed with Alexa Fluor 647-conjugated goat anti-rabbit (1:50, Invitrogen; A21245).

S100b and GFP dual IF staining were done sequentially as described above using anti-S100 (1:100, Dako; Z0628) with Alexa Fluor 568-conjugated goat anti-rabbit (1:50, Invitrogen; A11011) and Anti-GFP (1:50 Aves Labs Inc.; GFP-1010) with Alexa Fluor 647-conjugated donkey anti-chicken (1:50, Millipore; AP194SA6). A DAPI counterstain was then applied. Isotype negative control antibody-exposed slides were included as controls.

Single IF for specific histone modifications was carried out as above using rabbit monoclonal anti-methyl-histone H3 K27-3m (1:50, Cell Signaling; 9733S) and rabbit monoclonal anti-H2AK119u1 (1:100, Cell Signaling; 8240) primary antibodies.

## Additional files

**Additional file 1.** Weights at weaning and at the age of 3 months in animals with (Cre+) and without (Cre−) Nestin-Cre carrying Xist^2lox^ transgene (Xist^mut^/ Xist^2lox^) (upper panel) and in animals without Xist^2lox^ transgene (Xist^mut^/ Xist) (lower panel). Mice carrying Nestin-Cre exhibit weight reduction regardless whether they do or do not also carry Xist^2lox^ transgene (**p < 0.01, ***p < 0.001, Student’s *t* test).

**Additional file 2.** mRNA levels and DNA methylation of X-linked genes are altered upon Xist deletion. **A** MeCP2-EGFP mRNA levels measured by RT-qPCR in the RNA extracted from brains of Nestin-Cre Xist^mut^/MeCP2-GFP Xist^2lox^ females with Xist deletion in the brain (Cre+) relative to the MeCP2-EGFP mRNA level in MeCP2-EGFP/MeCP2-EGFP females that carry the reporter gene on the Xa (Xa) (*n* = 3 for each group, error bars indicate SD, *** *p* < 0.001, Student’s *t* test). **B** Scatter plot of RNA expression levels for genes on autosomes (black) and X-chromosome (red). The genes on X-chromosome exhibit an upward shift relative to autosomes. **C** Cumulative expression plots of fold expression changes in mRNA level among controls (Control 1/average (Controls 2 and 3) for genes on autosomes (black) and X-chromosome (red). The two curves are overlapping (*p* = 0.31, Wilcoxon rank-sum test). **D** Scatter plot of methylation levels in promoter proximal regions of the genes on autosomes (black) and X-chromosome (red). The genes on X-chromosome exhibit a downward shift relative to autosomes.

**Additional file 3.** Cumulative distribution plots of RNA-seq data of X-chromosome-linked genes compared to those on individual autosomes.

**Additional file 4.** Cumulative distribution plots of CpG island methylation of X-chromosome-linked genes compared to those on individual autosomes.

**Additional file 5.** Table 1: Primers used in the study.

## Abbreviations

ICM: inner cell mass
IF: immunofluorescence
IHC: immunohistochemistry
XCI: X-chromosome inactivation
Xi: inactive X-chromosome
Xm: maternal X-chromosome
Xp: paternal X-chromosome
