# Supplementary figures and images for: Perturbed maintenance of transcriptional repression on the inactive X-chromosome in the mouse brain after Xist deletion
Source: Epigenetics Chromatin. 2018 Aug 31;11:50. doi: 10.1186/s13072-018-0219-8 (PMC6118007; doi:10.1186/s13072-018-0219-8)

## Additional file 1

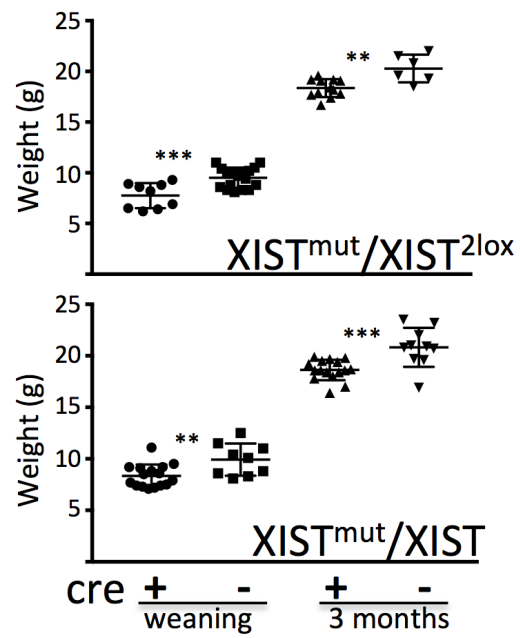

Supplement: Supplementary file 1 — Additional file 1. Weights at weaning and at the age of 3 months in animals with (Cre+) and without (Cre−) Nestin-Cre carrying Xist2lox transgene (Xistmut/ Xist2lox) (upper panel) and in animals without Xist2lox transgene (Xistmut/ Xist) (lower panel). Mice carrying Nestin-Cre exhibit weight reduction regardless whether they do or do not also carry Xist2lox transgene (**p < 0.01, ***p < 0.001, Student’s t test). [file 13072_2018_219_MOESM1_ESM.pdf]

Additional file 2

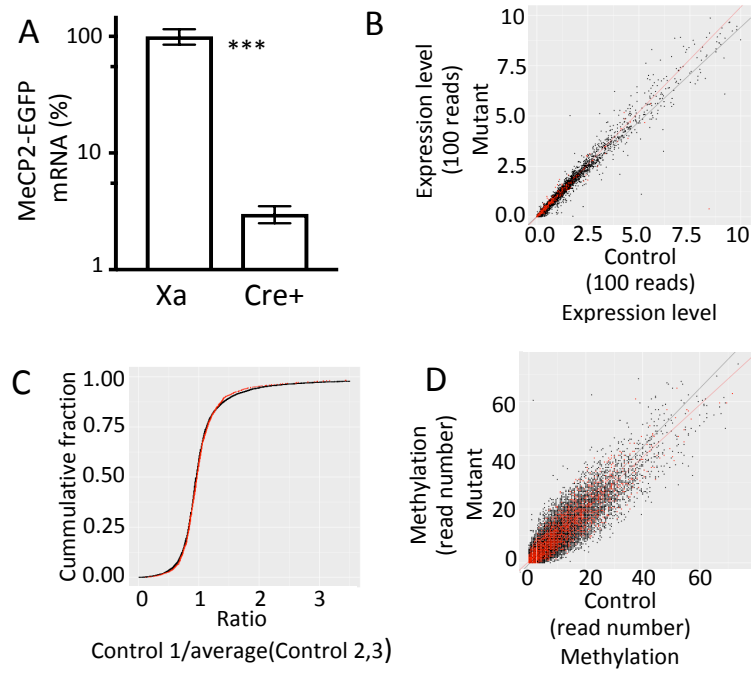

Supplement: Supplementary file 2 — Additional file 2. mRNA levels and DNA methylation of X-linked genes are altered upon Xist deletion. A MeCP2-EGFP mRNA levels measured by RT-qPCR in the RNA extracted from brains of Nestin-Cre Xistmut/MeCP2-GFP Xist2lox females with Xist deletion in the brain (Cre+) relative to the MeCP2-EGFP mRNA level in MeCP2-EGFP/MeCP2-EGFP females that carry the reporter gene on the Xa (Xa) (n = 3 for each group, error bars indicate SD, *** p < 0.001, Student’s t test). B Scatter plot of RNA expression levels for genes on autosomes (black) and X-chromosome (red). The genes on X-chromosome exhibit an upward shift relative to autosomes. C Cumulative expression plots of fold expression changes in mRNA level among controls (Control 1/average (Controls 2 and 3) for genes on autosomes (black) and X-chromosome (red). The two curves are overlapping (p = 0.31, Wilcoxon rank-sum test). D Scatter plot of methylation levels in promoter proximal regions of the genes on autosomes (black) and X-chromosome (red). The genes on X-chromosome exhibit a downward shift relative to autosomes. [file 13072_2018_219_MOESM2_ESM.pdf]

## Additional file 3

### RNA seq **X-chromosome** vs. autosomes

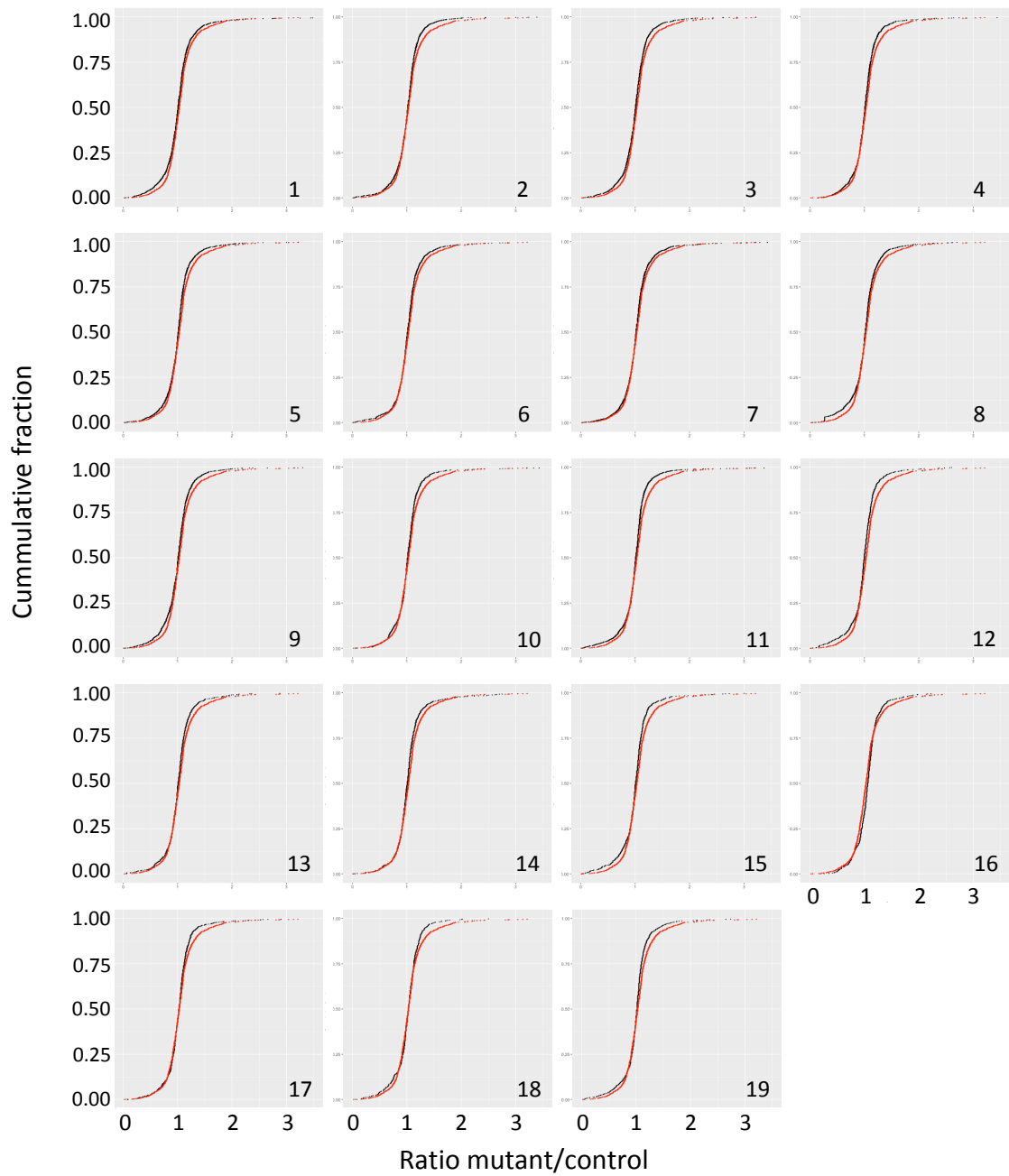

Supplement: Supplementary file 3 — Additional file 3. Cumulative distribution plots of RNA-seq data of X-chromosome-linked genes compared to those on individual autosomes. [file 13072_2018_219_MOESM3_ESM.pdf]

## Additional file 4

### Methylation X-chromosome vs autosome

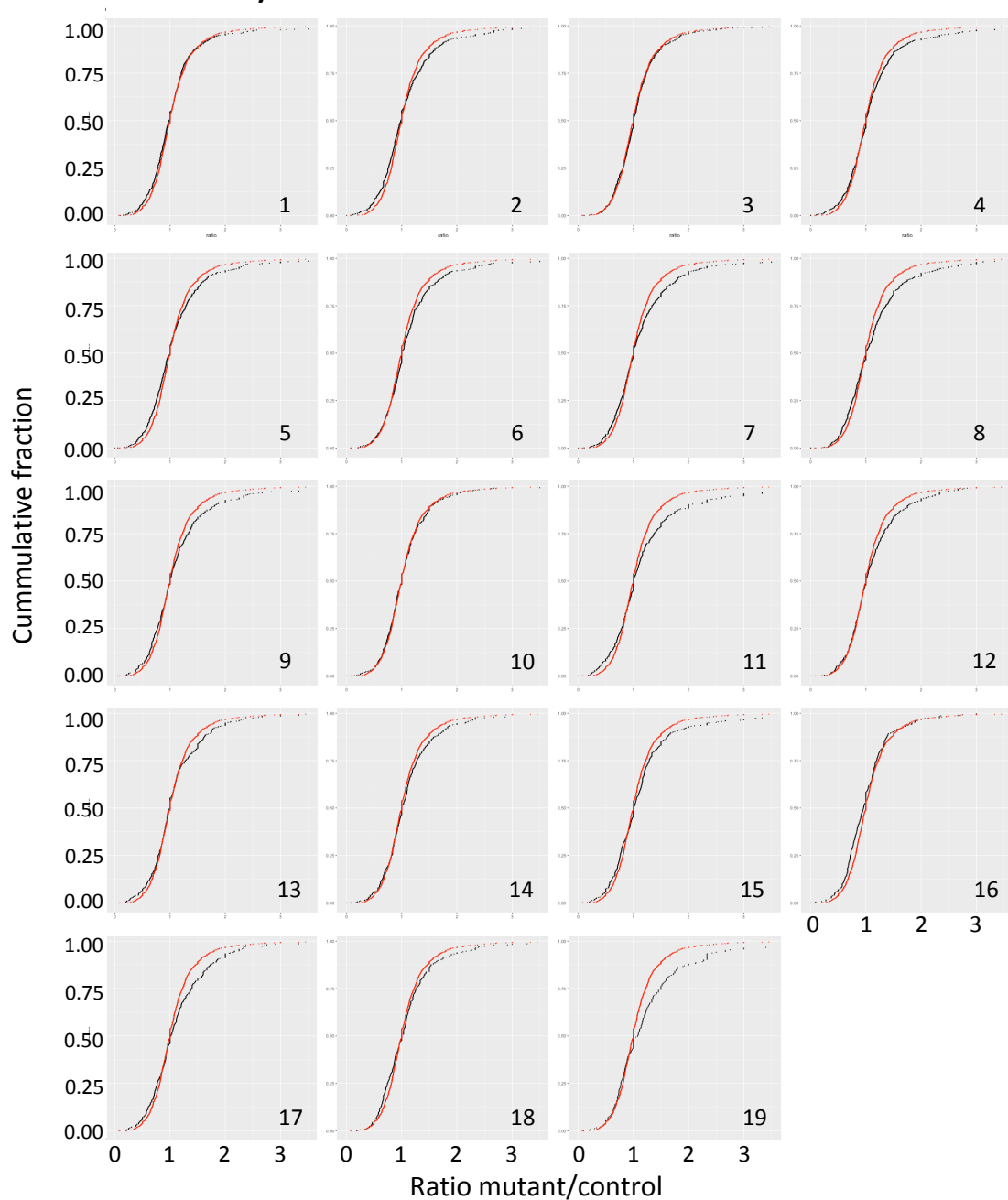

Supplement: Supplementary file 4 — Additional file 4. Cumulative distribution plots of CpG island methylation of X-chromosome-linked genes compared to those on individual autosomes. [file 13072_2018_219_MOESM4_ESM.pdf]
