# Supplementary material for: Perturbed maintenance of transcriptional repression on the inactive X-chromosome in the mouse brain after Xist deletion
Source: Epigenetics Chromatin. 2018 Aug 31;11:50. doi: 10.1186/s13072-018-0219-8 (PMC6118007; doi:10.1186/s13072-018-0219-8)
Supplement: Supplementary file 5 — Additional file 5. Table 1: Primers used in the study. [file 13072_2018_219_MOESM5_ESM.pdf]

Additional file 5

Table 1

Primers:
Xist2lox excision
F: 5’-CACTGGCAAGGTGAATAGCA-3’
R1: 5’TTTCTGGTCTTTGAGGGCAC-3’
R1 5’-ACCCTTGCCTTTTCCATTTT-3’

MeCP2-EGFP genotyping:
F: 5’-GCACCATCTTCTTCAAGGAC-3’
R: 5’-CCTTGATGCCGTTCTTCTG-3’

Nestin-Cre genotyping:
F: 5’-GTATGACGTCCCTTGCCATT-3’
R: 5’-ATGTCCATCAGGTTCTTGCG-3’

EGFP RT-qPCR:
F: 5’-ACCCTGAAGTTCATCTGCAC-3’
R: 5’-GGTCTTGTAGTTGCCGTCG-3’

GAPDH RT-qPCR
F: 5’-GCCCATCACCATCTTCCAG-3’
R: 5’-TTTGGCTCCACCCTTCAAG-3’
